# Chondrodysplasia Punctata: Early Diagnosis and Multidisciplinary Management of Conradi-Hünermann-Happle Syndrome (CDPX2)

**DOI:** 10.7759/cureus.75605

**Published:** 2024-12-12

**Authors:** Madjer Hatia, Diogo Roxo, Mafalda S Pires, Frederico Moeda

**Affiliations:** 1 Physical Medicine and Rehabilitation, Unidade Local de Saúde de Lisboa Ocidental, Lisbon, PRT; 2 Physical Medicine and Rehabilitation, Hospital de Cascais, Lisbon, PRT; 3 Rehabilitation Medicine, Hospital Dona Estefânia, Centro Hospitalar Universitário de Lisboa Central (CHULC), Lisbon, PRT

**Keywords:** chondrodysplasia punctata, conradi-hünermann-happle syndrome, rare genetic disease, rehabilitation, skeletal dysplasias

## Abstract

Chondrodysplasia punctata (CP) is a rare skeletal dysplasia characterized by punctate calcifications in areas of endochondral ossification, with Conradi-Hünermann-Happle syndrome (CDPX2) being the most common form. This study presents a clinical case of a 10-month-old female child, diagnosed with CDPX2 following a referral from a neonatology department of a secondary hospital center to a genetics consultation at a tertiary hospital center in Portugal. Despite normal prenatal monitoring, postnatal evaluations revealed typical manifestations of the syndrome, including nasomaxillary hypoplasia, macrocephaly, and skeletal abnormalities confirmed through imaging. Genetic testing using whole exome sequencing (WES) based on next-generation sequencing (NGS), targeting a panel of genes associated with skeletal dysplasia, revealed a loss-of-function variant in the emopamil-binding protein (EBP) gene. The child received multidisciplinary care from a team composed of orthopedy, dermatology, and physical medicine and rehabilitation doctors, aimed at promoting motor development and managing the condition's complexities. This study underscores the importance of early diagnosis and a comprehensive treatment approach to enhance the quality of life for individuals with CP, while also highlighting the need for increased awareness of such rare genetic disorders among healthcare professionals. Ongoing research into genetic therapies offers hope for future advancements in treatment options.

## Introduction

People with chondrodysplasia punctata (CP) have a rare type of skeletal dysplasia identified by punctate calcifications in areas of endochondral ossification. Most often, one can find these in the vertebrae, ribs, epiphyses of long bones (epiphyseal dysplasia), or even in the trachea [1].

This group of pathologies with genetic etiology is classified based on the transmission type and heredity. In this way, we may be dealing with an autosomal dominant form, an autosomal recessive or rhizomelic form, or forms linked to the X chromosome recessive (CDPX1) and dominant (CDPX2 or Conradi-Hünermann-Happle). Scientists described the following two milder forms in the 1990s: tibia-metacarpal and the brachycephalic. Conradi-Hünermann-Happle syndrome is the most common form of punctate chondrodysplasia. A change in the gene for "emopamil-binding protein" (EBP), which produces EBP, also known as the 3-beta-hydroxysteroid-Delta(8),Delta(7)-isomerase, causes this X-linked dominant disorder [2].

Being extremely rare, it has been a challenge for doctors and researchers to estimate their prevalence accurately. Med-Life Discoveries (MLD), in partnership with a leading genetic epidemiology company, Leiden Analytics, estimated the disease burden in the United States and Europe through a genetic epidemiological model. An estimated 2.4 million people in the United States alone carry mutations that can cause CP. The incidence of births with CP was 14-23 births per year in the United States and 18-28 births in Europe, which translates to 0.5-0.7 cases of CP per 100,000 births. CP exhibits significant clinical variability, including very mild forms where the phenotypic manifestations are subtle and may only become apparent in adulthood. Due to subtle symptoms, many individuals remain undiagnosed or are misdiagnosed as the clinical features may not be distinctive enough. Some may present with only a few features of the syndrome but still carry the pathogenic EBP gene mutation, which is why genetic testing is crucial for accurate diagnosis. The severity of clinical manifestations varies based on factors such as the specific genetic mutation, the allelic expression of the mutated gene, and the individual's overall genetic background, including potential modifier genes [3,4].

This mutation is generally lethal in males, with an estimated 95% of patients being female. However, there are some published case reports of male patients with manifestations of the disease [5,6]. This disease shows up in the form of birth defects in the bones, like rhizomelic micromelia in the humerus and femur, short stature, clinodactyly, camptodactyly, hip dysplasia, joint stiffness, clubfoot, and growth delay. There are often problems with the face, such as frontal bossing, malar hypoplasia, anteverted nostrils, and dysplastic ears. There are also changes in the spine (such as kyphoscoliosis), the eyes (including microphthalmia, microcorneas, and cataracts), hair loss with cicatricial alopecia patches, and changes in the skin and limbs, such as erythrodermic ichthyosis. These changes usually resolve during childhood, but they can evolve into follicular atrophodermatitis. This pathology can progress with hyper or hypopigmentation of the skin [7,8]​​​.

Ultrasound enables the prenatal diagnosis of this pathology by detecting skeletal asymmetries, growth delay, and polyhydramnios. Molecular prenatal diagnosis is also possible through the analysis of chorionic villi or amniocentesis [9].

Following birth, clinical and radiological characteristics support the suspicion of this diagnosis, and biochemical findings should confirm it. Therefore, there are the following two distinct ways to diagnose it: (1) analysis of plasma sterol with markedly elevated levels of 8(9)-cholestenol and 8-dehydrocholesterol and (2) detection of a missense mutation (c.307G>A; p.E103K) in emopamil, the gene of the human sterol isomerase binding protein 12 [8].

The therapeutic program for patients with this pathology is multidisciplinary. Affected patients usually require dermatological care, ophthalmological care, orthopedic corrections for skeletal anomalies, and frequent follow-up in Physical Medicine and Rehabilitation (PMR) aimed at gaining functionality and greater autonomy in daily tasks [10].

## Case presentation

A 10-month-old female child, suspected of having punctate chondrodysplasia, was referred by the neonatology department of a secondary hospital to a genetics consultation at a tertiary hospital in Portugal.

The medical record revealed no family history of short stature or other skeletal dysplasias. The pregnancy was monitored, and no complications were found, except for the breech presentation. The delivery occurred at 38 weeks and four days via cesarean section, with somatometric measurements appropriate for gestational age and sex. The pregnancy monitoring tests and ultrasound did not reveal any abnormalities.

As the standard procedure for checking for hip dysplasia in breech presentations, an ultrasound of both hip joints showed punctiform calcifications in the bone epiphyses. This led to a referral for multidisciplinary consultations at Hospital Dona Estefânia (HDE).

The child underwent initial evaluation in the genetics consultation, where genetic testing using whole-exome sequencing (WES) based on next-generation sequencing (NGS), targeting a panel of genes associated with skeletal dysplasia, identified a heterozygous loss-of-function (LoF) variant in the emopamil-binding protein (EBP) gene. This finding confirmed the diagnosis of Conradi-Hünermann-Happle syndrome, X-linked type 2 punctate chondrodysplasia (CDPX2; EBP gene). Genetic counseling was also provided to the parents, taking into account the possibility of a maternal de novo origin or paternal germline mosaicism in this situation. The couple was provided with a carrier study to assess the risk of recurrence, and they were informed that this child carries 50% risk of transmission to any future offspring, with clinical manifestations varying based on sex and potential mosaicism. There was proposed genetic counseling at the age of majority or during the parental project. At the time of the PMR consultation, the 10-month-old child presented with nasomaxillary hypoplasia, characterized by a depressed nasal bridge and inwardly turned nostrils. The child also presented with macrocephaly, alopecia, hyperkeratotic lesions on the scalp, and outward rotation of the lower limbs. Regarding psychomotor development, the child exhibited effective head control although she still could not maintain sitting independently.

**Figure 1:**
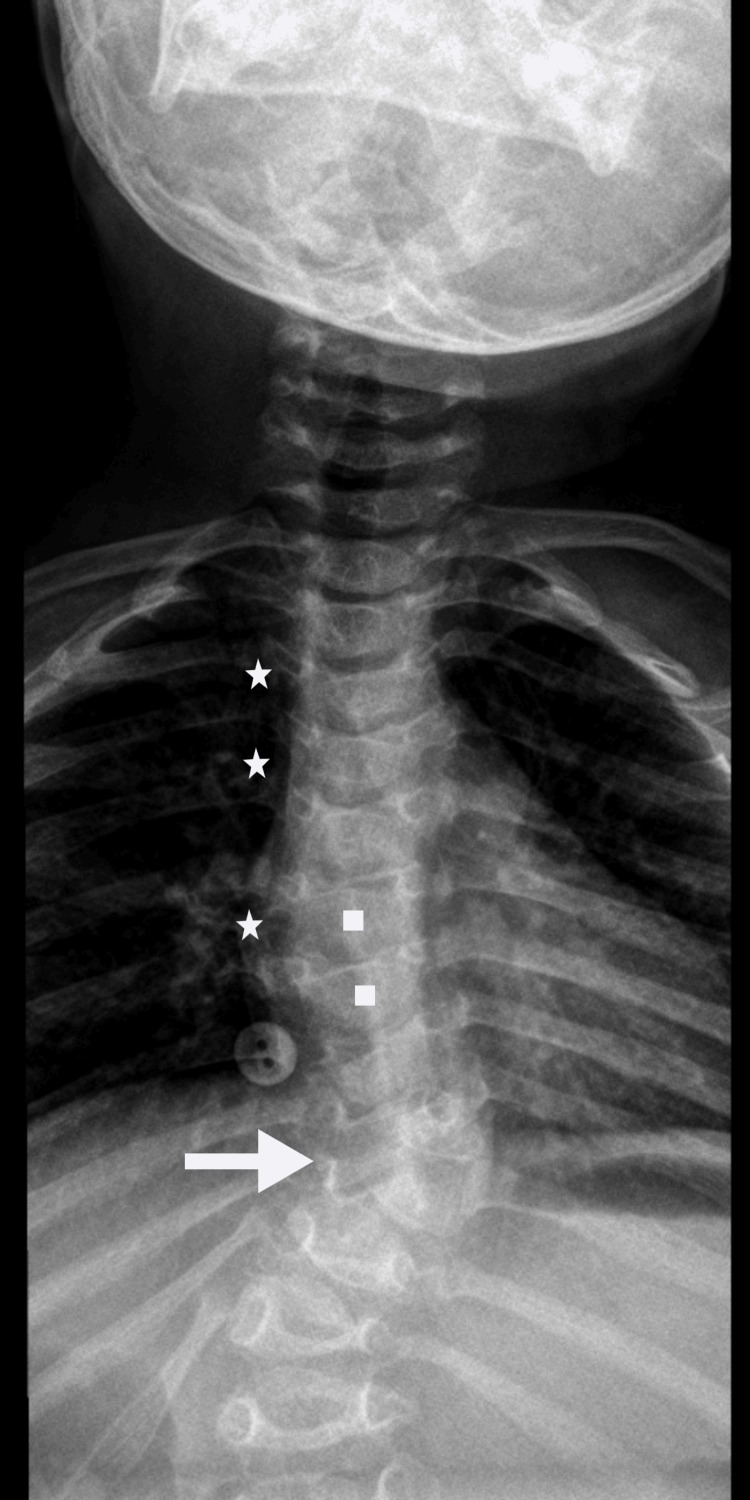
Spine radiography of the patient. The image shows calcifications of the epiphyseal cartilages in the anterior costal arches and the vertebral column (stars), scoliosis (arrow), and vertebral dysmorphisms, such as reduced height of vertebral bodies and rounded morphology of vertebral bodies at the dorsolumbar level (squares).

A simple X-ray of the skeleton revealed calcifications in many of the epiphyseal cartilages of the axial and appendicular skeletons, especially near the anterior costal arches and the vertebral column. The patient presented with dysmorphic ribs ("oar-shaped ribs"), spinal dysraphism at C4, scoliosis, and vertebral dysmorphisms, such as reduced vertebral body height, hemivertebra at D9, and rounded morphology of some vertebral bodies at the dorsolumbar level (Figure 1).

**Figure 2:**
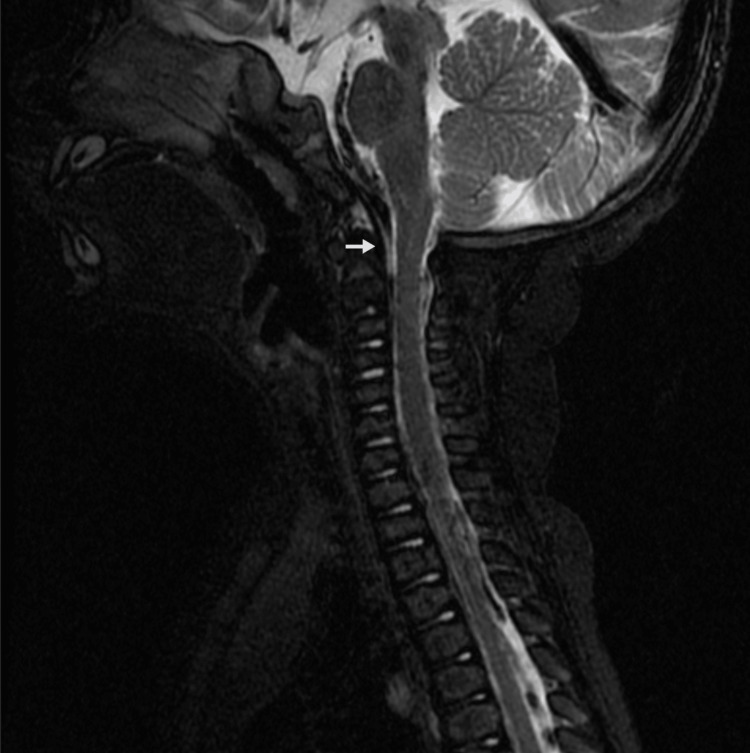
Cervical spine MRI (sagittal view) of the patient. The image shows a reduction in the caliber of the occipital foramen but without signs of medullary parenchymal molding (arrow).

The femoral neck got shorter and wider, the ossification of the proximal femoral epiphyses took a little longer than expected, and the distal femur turned outward and had bilateral genu varum. A brain and cervical spine MRI showed changes in the shape of the basiocciput, the body of C2, and the somatic parts of C3 and C4. It also showed that the perichondral spaces of the ossification centers were hardened. These changes resulted in a significant reduction in the caliber of the occipital foramen, without any signs of medullary parenchymal molding (Figure 2).

The child began a tailored rehabilitation program designed to support and encourage normal motor development. The program includes physical therapy to enhance muscle strength, joint mobility, and coordination, with a focus on developing basic gross motor skills, such as rolling, crawling, and eventually standing or walking. The therapy includes age-appropriate activities that encourage movement and flexibility, with gentle stretching exercises to promote range of motion and reduce discomfort. Occupational therapy was also incorporated to promote fine motor skills, such as grasping toys, reaching, and improving hand-eye coordination. Internal rotation straps for the lower limbs and knee stabilizing orthoses were prescribed together with a standing frame for orthostatic training. Her subsequent follow-up in PMR consultations prioritized the acquisition of psychomotor skills and the improvement of axial and appendicular alignment, facilitating the primary objectives. She has maintained ongoing follow-up through regular consultations in the orthopedics and dermatology department, with no interventions required from either specialty at this stage. The periodic assessment was carried out according to the key stages of child development.

## Discussion

In this study, the child was genetically diagnosed with Conradi-Hünermann-Happle syndrome and is female, which corresponds to the most common form of the disease and the most affected sex, respectively [2,5]. Although it is possible to make an imagiologic prenatal diagnosis by detecting skeletal asymmetries, growth delay, and polyhydramnios or molecular prenatal diagnosis through the analysis of chorionic villi or amniocentesis, the screening did not identify any changes [9].

As acknowledged, the confirmation of the diagnosis is through biochemical findings of elevated levels of 8(9)-cholestenol and 8-dehydrocholesterol or by molecular genetic testing [8]. Genetic analysis revealed a heterozygous LoF variant in the EBP gene (MIM#302960 {Mendelian Inheritance in Man}, ORPHA:35173 {Orphanet identifier}), which encodes 3-beta-hydroxysteroid-Delta(8),Delta(7)-isomerase, an enzyme critical for cholesterol biosynthesis. Disruption of this enzyme leads to the accumulation of sterol precursors, resulting in the characteristic phenotypic manifestations of this X-linked dominant disorder. The pathogenicity of loss-of-function variants in EBP has been substantiated by functional studies demonstrating elevated levels of 8-dehydrocholesterol and cholest-8(9)-en-3beta-ol in affected individuals [11]. Additionally, numerous case reports have documented the association between EBP mutations and CDPX2, further reinforcing this genotype-phenotype correlation [12]. Specific details such as the genomic location, nucleotide change, and protein change were not provided. The confirmation of the diagnosis enabled appropriate genetic counseling for the parents, addressing the hereditary implications and the potential risk of recurrence in future pregnancies.

This disease shows up in the form of birth defects in the bones, with often problems with the limbs, face, spine, eyes, hair, and skin [7,8]. This child presented typical clinical manifestations of the disease, on the most affected areas as described, such as nasomaxillary hypoplasia, anteverted nostrils, macrocephaly, alopecia, hyperkeratosis lesions on the scalp, lower limbs in external rotation, and lumbosacral hyperlordosis, as well as typical radiological findings such as punctate calcifications in the epiphyseal cartilages of the appendicular and axial skeleton, dysmorphic ribs, spina bifida, vertebral dysmorphisms, rhizomelic shortening of long bones, dimensional asymmetry between the bones of both limbs, shortening and widening of the femoral neck, and bilateral genu varum.

While many skeletal dysplasias involve cervical lesions resulting from instability or spinal cord compression, such findings are rare in chondrodysplasia punctata (CP). CP is typically considered a distinct group of skeletal dysplasias with unique genetic, metabolic, or developmental causes, and it is not commonly associated with other skeletal dysplasia syndromes. Cervical spinal stenosis can cause subtle and easily overlooked symptoms, but it is a potentially serious condition [10]. The child underwent an MRI scan that showed basioccipital dysmorphism with a reduction in the caliber of the foramen magnum but without evidence of myelopathy.

The affected patients usually require multidisciplinary care with dermatological, ophthalmological, and orthopedic contributions and close follow-up in PMR in order to gain the best possible functionality and autonomy in daily tasks [10]. The child, who presents various clinical and radiological manifestations that are typical of the syndrome, is followed by a multidisciplinary team with interventions aimed at promoting motor and functional development. While there is no cure for chondrodysplasia punctata, treatment focuses on managing symptoms and improving quality of life [10].

## Conclusions

The presented clinical case illustrates the complexity and challenges associated with the diagnosis and management of punctate chondrodysplasia, specifically Conradi-Hünermann-Happle syndrome (CDPX2). The importance of early diagnosis and multidisciplinary follow-up is fundamental to improve the quality of life and autonomy of the affected child, highlighting the need for greater awareness of these rare conditions among healthcare professionals.

Advances in genetic testing have allowed for earlier diagnosis, leading to more proactive management of symptoms. Additionally, researchers are exploring potential gene therapies that could target the specific genetic mutations responsible for the disorder. These promising developments offer hope for improved outcomes and quality of life for individuals affected by chondrodysplasia punctata.
